# Glucose Improves the Efficacy of Photobiomodulation in Changing ATP and ROS Levels in Mouse Fibroblast Cell Cultures

**DOI:** 10.3390/cells12212533

**Published:** 2023-10-27

**Authors:** Jaimie Hoh Kam, John Mitrofanis

**Affiliations:** 1 Fonds de Dotation Clinatec, Grenoble Alpes University, 38000 Grenoble, France; john.mitrofanis@me.com; 2 Institute of Ophthalmology, University College London, London EC1V 9EL, UK

**Keywords:** cell death, infrared, cell culture, mitochondria, metabolism, fibroblasts

## Abstract

In this study, we tested the idea that photobiomodulation—the application of red to near infrared light (~λ = 600–1300 nm) to body tissues—is more effective in influencing cell metabolism when glucose is readily available. To this end, we used a mouse fibroblast (L-929) cell culture model and had two sets of conditions: non-stressed (10% FBS (foetal bovine serum)) and stressed (1% FBS), both either with or without glucose. We treated (or not) cells with photobiomodulation using an 810 nm laser at 1.5 mW/cm^2^ (~0.72 J/cm^2^). Our results showed that photobiomodulation was neither cytotoxic nor effective in enhancing measures of cell viability and proliferation, together with protein levels in any of the cell cultures. Photobiomodulation was, however, effective in increasing adenosine triphosphate (ATP) and decreasing reactive oxygen species (ROS) levels and this was—most importantly—only in conditions where glucose was present; corresponding cultures that did not contain glucose did not show these changes. In summary, we found that the benefits of photobiomodulation, in particular in changing ATP and ROS levels, were induced only when there was glucose available. Our findings lay a template for further explorations into the mechanisms of photobiomodulation, together with having considerable experimental and clinical implications.

## 1. Introduction

Many previous studies have reported that photobiomodulation—the application of red to near infrared light (~λ = 600–1300 nm) to body tissues—can generate a range of beneficial cellular effects in many different types of body-wide disorders, from neurological [1,2,3,4] to microbial [5], and from cardiovascular [6] to musculoskeletal [7] and dermatomal [8].

The precise photobiomodulation-induced mechanisms that generate these beneficial outcomes in cells are not entirely clear, although a central target is the mitochondria. Many authors have reported that these organelles have photoacceptors that absorb light across a wide range of wavelengths in the red to near infrared spectrum, from 630–660 nm [9,10,11] to 808 nm, and 980 nm [12,13] to 1064 nm [14]. The most well-known of these photoacceptors is cytochrome c oxidase, the terminal enzyme of the mitochondrial respiratory chain. After absorbing the red and near infrared light, electrons are excited from the ground state, resulting in a higher mitochondrial membrane potential and an increase in the activity of the enzyme adenosine triphosphate (ATP) synthase that produces more ATP energy for the cell. Signalling molecules are produced, including a brief burst of reactive oxygen species (ROS), promoting nitric oxide release and an increase in the expression of genes related to protein synthesis, cell migration and proliferation, anti-inflammatory signalling, antiapoptotic proteins, and antioxidant enzymes [2,15,16]. It should be noted that, under pathological conditions, for example after hypoxia, trauma, or neurodegenerative disease, photobiomodulation has also been shown to reduce the toxic overload of ROS that is produced by the damaged and dysfunctional mitochondria, and hence helps to reduce the distress and subsequent death of cells [2,17]. In recent years, it has become clear that other photoacceptors are also present in the cell [18], and interfacial nanowater has been suggested to be a likely candidate [16,19]. Layers of nanowater are found within the mitochondria and these tend to become viscous; the increase in viscosity can impede the efficient function of ATP synthase, leading to cell distress. Photobiomodulation has been related to a decrease in the viscosity of nanowater, leading to an increase in the activity of ATP synthase and the subsequent production of more ATP [16,19].

Hence, through an increase in ATP after mitochondrial activation, photobiomodulation can improve energy metabolism within cells. But, in order to activate the mitochondria to produce the ATP effectively, there should be sufficient cellular fuel available for the mitochondria to work with. Glucose is such a fuel, and it can be made available to cells from two main sources [20,21]. First, glucose can be transported into the cells directly from the blood supply by glucose transporters and then converted to pyruvate [22,23]. When there is oxygen available, the pyruvate enters the mitochondria to undergo aerobic respiration. This activates ATP synthase, and thirty-six molecules of ATP are produced [24]. When there is less oxygen available and/or under periods of high energy demand, the pyruvate undergoes anaerobic respiration and there is a rapid conversion to ATP, with the end-product being lactate and only two ATP molecules [25]. This is the process of glycolysis. Second, during times of either low blood glucose levels or in cases of extreme need, glucose can be used from glycogen storage [26]. The main sites of glycogen storage are within the muscles, the liver, and the brain [27].

In this study, we tested the idea that, without glucose, the effectiveness of photobiomodulation in influencing cellular metabolism is compromised severely (e.g., [28,29]). To this end, we explored the impact of photobiomodulation on mouse fibroblasts in cell cultures, with and without the availability of glucose. We chose to use fibroblasts because they are relatively simple cells and easy to grow in culture; they are the major cell active in connective tissue and produce many extracellular matrix proteins such as collagen, glycosaminoglycans, and proteoglycans [30]. Our results will provide further insights into the mechanisms behind photobiomodulation, together with having a clear impact on how photobiomodulation treatment is used in both the experimental laboratory and in the clinic.

## 2. Materials and Methods

### 2.1. Cell Cultures and Experimental Outline

Mouse fibroblast L-929 (CCL-1) (ATCC) were grown in Eagles Minimum Essential Medium (EMEM) containing Earle’s balanced salts and sodium bicarbonate (Sigma, Saint Quentin Fallavier, France), supplemented with 1% L-Glutamine (Gibco Life Technologies, Illkirch, France) and 10% fetal bovine serum (FBS) (Avantor, Rosny-sous-Bois, France). Cell cultures were incubated at 37 °C in 5% CO_2_ with 85% humidity.

We tested the effect of photobiomodulation on culture media that have been considered to generate a non-stressed, so-called “normal” environment for the cells (in 10% FBS), either with or without glucose. Next, we explored the effect of photobiomodulation on cells in a more stressed environment, containing fewer nutrients (in 1% FBS), again either with or without glucose [31,32,33]. Specifically, we had four main groups of cell cultures:

(1) Non-stressed (10% FBS) with glucose (~1 g/L of glucose);
(2) Non-stressed (10% FBS) without glucose;
(3) Stressed (1% FBS) with glucose (~1 g/L of glucose);
(4) Stressed (1% FBS) without glucose.

In this study, the basic medium consisted of EMEM with 1% L-Glutamine. All the media were phenol free. It should be noted that the commercial FBS serum (Gibco) did contain very small traces of glucose (1.32 mg/L in the 10% FBS and 0.132 mg/L in the 1% FBS), but these traces were considered negligible. Overall, the net level of glucose in groups 2 and 4 were considerably lower than in groups 1 and 3, certainly enough for us to gauge an experimental effect.

### 2.2. Photobiomodulation Treatment

Cell cultures were exposed to a diode laser emitting continuous near infrared light (λ = 810 nm) with a beam diameter of 2 mm (Ti-sapphire Laser). The laser was positioned on top of the polystyrene transparent cell culture six-well plate (13 × 10 cm), at a distance of approximately 10 cm. Following Sharma and colleagues [34], who used an 810 nm laser to treat neurones at fluences from 0.03–30 J/cm^2^ on cortical neurones and obtained cellular responses from 0.3 J/cm^2^, the dose here was 1.5 mW/cm^2^ (measured at the level of the cells in the dish) for 120 s (total of 0.72 J/cm^2^), and the cells were light-treated twice per day (morning and afternoon) over 24 h for two days. To prevent nuisance variables from other light sources, laser exposure took place in the dark.

Assays were conducted either within 1 h (short-term) or within 18 h (longer-term) following the final photobiomodulation treatment. For controls, a matching series of cell cultures were not exposed to photobiomodulation.

We had four measures of the impact of photobiomodulation on our four groups of cultures: cell viability, cell proliferation and protein content, cell stress (ROS assay), and metabolic activity (ATP assay) at both 1 h (short-term) and 18 h (longer-term) after the last photobiomodulation treatment (or not).

### 2.3. Cell Viability Test

An MTS/PMS (3-(4,5-dimethylthiazol-2-yl)-5-(3-carboxymethoxyphenyl)-2-(4-sulfophenyl)-2 H-tetrazolium/phenazine methosulfate) test (Promega, Charbonnieres-les Bains, France) was performed to evaluate the impact of photobiomodulation treatment on the fibroblast (L929) cells. This assay, which measures cell viability (i.e., cellular metabolic activity and proliferation), is based on the reduction in MTS/PMS reagents to produce soluble formazan products by viable and metabolically active cells. The decrease in the number of viable cells may be caused by one of two processes: actual cell death (cytotoxic effect) or inhibition of cell metabolism and/or proliferation (cytostatic effect). Cells were plated in quadruplicate in a 96-well plate at a density of 10,000 cells/well. After the last photobiomodulation treatment, 20 µL of the MTS/PMS mixture was added to the cell medium and cells were incubated for 3 h at 37 °C in 5% CO_2_ with 85% humidity. After the 3 h, the media containing MTS/PMS was removed and transferred into another 96-well plate and absorbance was measured at 492 nm using the FLUOstar Omega (BMG Labtech, Champigny-sur-Marne, France) according to the instructions of the manufacturer.

Cells were then plated in either triplicate or quadruplicate in 6-well plates at a density of 150,000 cells/well in normal medium for 24 h; then, the medium was changed to the respective media conditions and cells were counted either within 1 h following the final treatment or 18 h later. Cells were detached using Trypsin-EDTA 0.25% (Fisher Scientific, Illkirch, France). The cells were collected and prepared for the different assays below.

### 2.4. Cell (Number) Proliferation and Protein Measurement

Cell number (proliferation) was determined by using trypan blue exclusion assay, which is based on the principle that living cells have intact cell membranes that will exclude the trypan blue and remain colourless, while dead cells will take up the blue staining. The cells in all four different groups that were either photobiomodulation or non-treated were counted using the TC20 counting machine.

Trypan blue (Thermo Fisher Scientific) staining was used to determine the approximate percentage of viable cells in the samples. Equal volumes of trypan blue dye (Gibco Life Technologies) and cell suspension were pipetted and allowed to stain briefly. The mixture was then loaded into a TC20 counting slide (Bio-Rad, Marnes-la-Coquette, France) and the latter was inserted into the TC20 instrument (Bio-Rad, Marnes-la-Coquette, France). A gating of 10–22 µm was selected as optimal for viable cells. Non-viable cells exhibit blue stain due to damaged or compromised cell membranes as the dye enters the cells. Viable cells with undamaged cell membranes remain clear.

Measurement of the total protein level was performed using a commercially available kit (BCA Protein Assay by Thermo Scientific). Bovine serum albumin was used as a standard, and the amount of protein was measured following the instructions of the manufacturer.

### 2.5. Reactive Oxygen Species (ROS) Assay

To explore the level of mitochondrial stress in the fibroblasts, we measured the quantity of intracellular ROS using the dichloro-dihydrofluorescein diacetate (DCFH-DA) method. ROS are produced in the mitochondria as a by-product of oxidative phosphorylation. When cells are damaged, mitochondria produce an excessive amount of ROS which leads to cell stress and inflammation and can result in cell death (see Introduction).

Cells were harvested and homogenised in 200 µL of ice-cold 0.4 M Tris-HCl buffer (pH 7.4) (Merck, St Quentin Fallavier, France), followed by a 10 min centrifugation at 2000× *g* at 4 °C. 50 µL of each supernatant was added to a 96-microplate well containing 7.5 µL of 5 µM DCFH-DA (Merck, St Quentin Fallavier, France), and total volume was adjusted to 100 µL with homogenizing buffer. After 1 h incubation at room temperature, the conversion of DCFH-DA to DCF was measured at 485 nm excitation and 520 nm emission. The protein concentration of each sample was measured using the BCA method (Fisher Scientific, Illkirch, France).

### 2.6. Adenosine Triphosphate (ATP) Assay

Intracellular ATP production was quantified using the ATP Determination kit (Fisher Scientific, Illkirch, France). Cells were harvested and homogenised in 100 µL of 6 M guanidine-HCl in extraction buffer (100 mM Tris and 4 mM EDTA, pH 7.8) to inhibit ATPases, followed by freezing in dry ice. The homogenate was then heat treated at 95 °C for 5 min and then centrifuged for 15 min at maximum speed (13,500 RPM). The supernatant was collected, and ATP was measured according to the manufacturer’s instructions. The protein concentration of each sample was measured using the BCA method (Thermo Scientific, Illkirch, France).

### 2.7. Statistics

The data were analysed with GraphPad Prism v10 (San Diego, CA, USA) and statistical analysis was performed using a two-way ANOVA with Tukey’s multiple comparisons as a post-hoc test and two-tailed Mann–Whitney U test to compare between the two groups. Data are shown as the mean ± SEM and expressed as the mean percentage (%) of control values. The significance was asserted as * *p* < 0.05, ** *p* < 0.01, *** *p* < 0.001, and **** *p* < 0.0001.

## 3. Results

**Figure 1:**
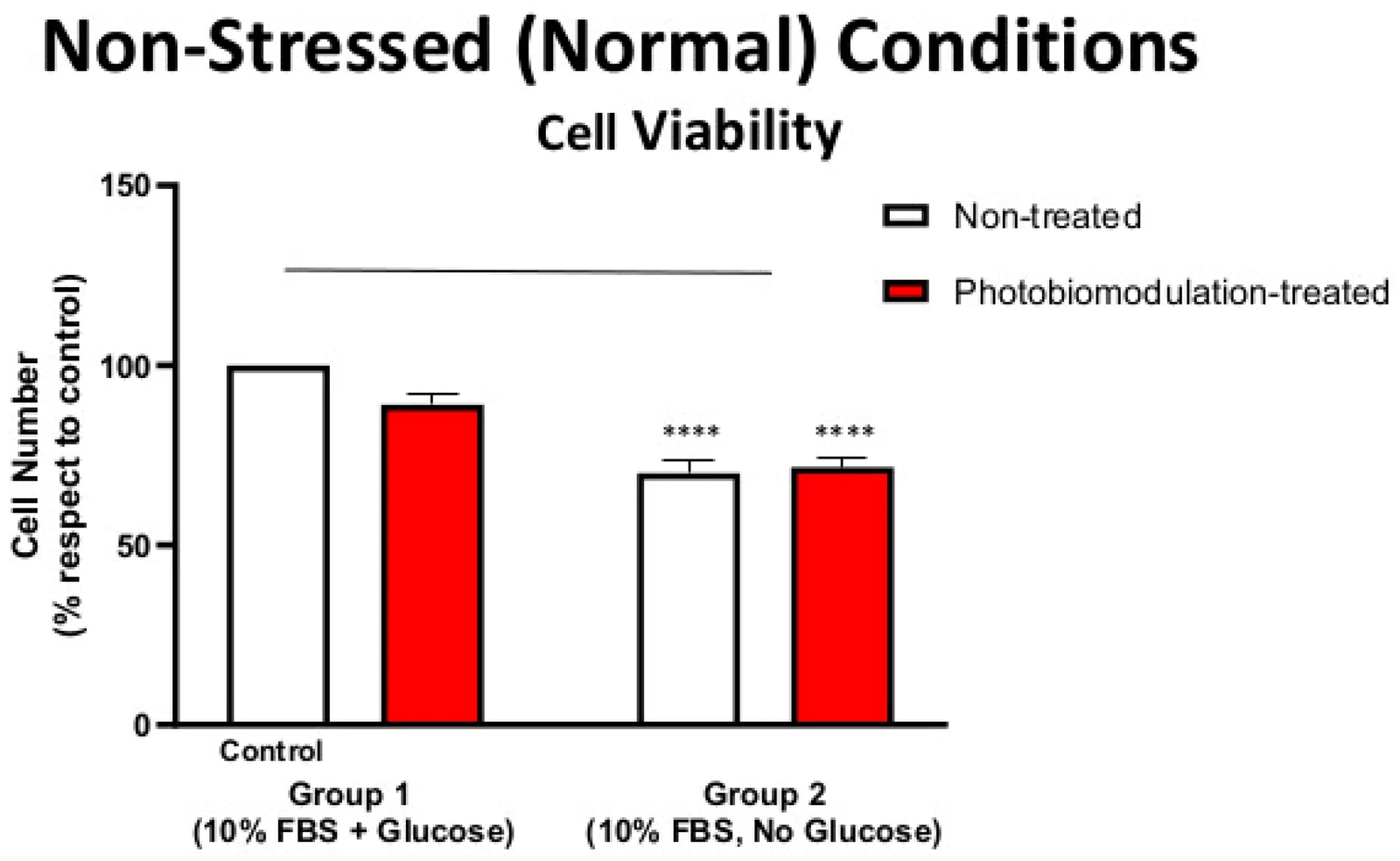
Graph showing the effect of photobiomodulation on the cell viability of cultures of fibroblast cells under non-stressed (normal) conditions, with or without glucose. The results show that there is a statistically significant decrease in groups 2 (*p* < 0.0001; both photobiomodulation-treated (in red) and non-treated (in white)) compared to the control (group 1). The results also show that photobiomodulation treatment had no effect and was not detrimental to the fibroblast cells (*n* = 12). The comparison between the control group cell cultures (group 1; non-treated cells) and the photobiomodulation- and non-treated cells of group 2 is shown by the line above each of the columns; if the line finishes in-between the columns, then it reflects the same level of statistical significance. Non-significant comparisons are not indicated. Data are expressed as the mean percentage (%) of control values (group 1; non-treated cells) ± SEM, **** *p* < 0.0001, and this applies to all of the non-stressed (normal) conditions.

**Figure 2:**
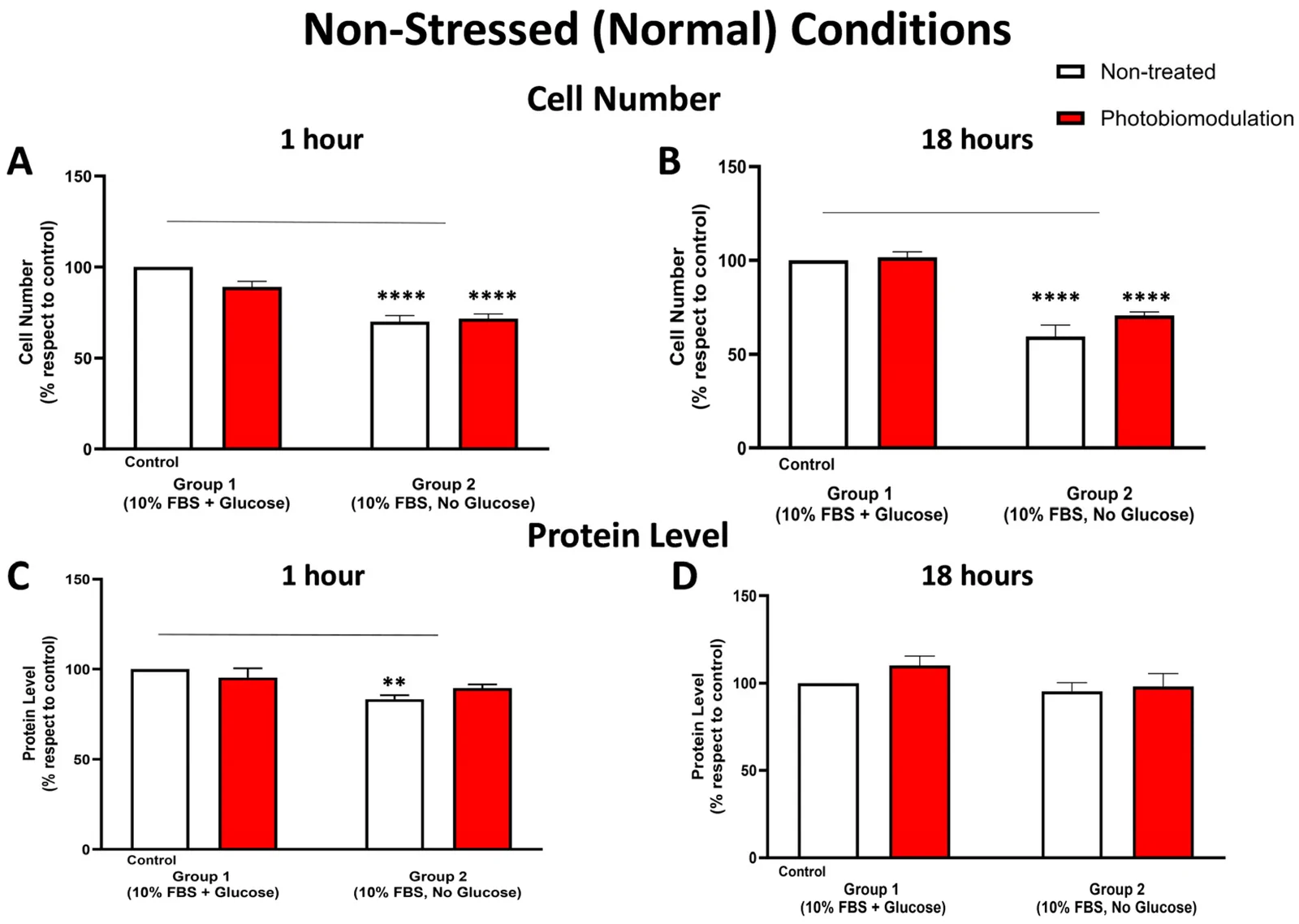
Graphs showing the effect of photobiomodulation on cell (number) proliferation at 1 h (**A**) and 18 h (**B**), respectively, post-photobiomodulation treatment in non-stressed conditions. At both 1 h and 18 h post-treatment, there were statistically significant reductions in the number of cells, both in the treated (in red) (*p* < 0.0001 for both 1 h and 18 h) and non-treated groups (in white) (*p* < 0.0001 for both 1 h and 18 h) in group 2, when compared to the control cells in group 1. There were no significant differences in the photobiomodulation-treated cells compared to the non-treated cells (*p* > 0.5 for both groups and for both time points; *n* = 9). (**C**,**D**) are graphs showing the level of protein within 1 h (**C**) and 18 h (**D**) after the last application of photobiomodulation. There was a significant decrease in the protein level (**C**) of the fibroblast cell cultures without glucose (non-treated) from group 2 (*p* < 0.01) compared to the control in group 1, but this decrease was not statistically significant between the photobiomodulation-treated group in group 2 and the control in group 1 (*p* > 0.5) (*n* = 12 for each group). After 18 h post-treatment (**D**), there were no significant differences in protein levels either between cell cultures with and without glucose or between non-treated and treated cells (*p* > 0.5, *n* = 9 for each group). The comparison between the control group cell cultures (group 1; non-treated cells) and the photobiomodulation- and non-treated cells of group 2 is shown by the line above each of the columns; if the line finishes in-between the columns, then it reflects the same level of statistical significance. Non-significant comparisons are not indicated. ±SEM, ** *p* < 0.01 and **** *p* < 0.0001.

**Figure 3:**
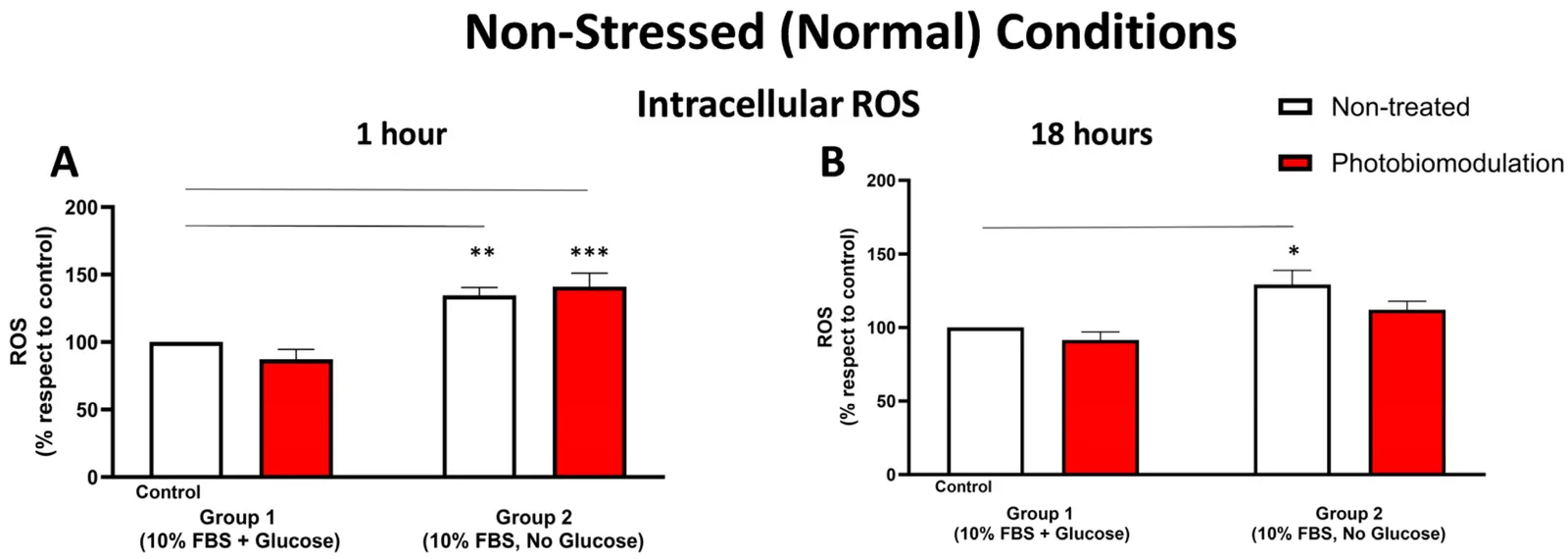
Graphs showing the level of cellular stress at 1 h (**A**) and 18 h (**B**) in non-stressed conditions, determined by measuring the level of ROS using the DCFH-DA method. After 1 h post-treatment (**A**), there was a significant increase in group 2, in both the photobiomodulation-treated (in red) (*p* < 0.001) and non-treated (in white) (*p* < 0.01) cells, when compared to the control in group 1. There was no significant difference between non-treated and photobiomodulation-treated cells in groups 1 and 2 (*n* = 12 for each group). After 18 h post-treatment (**B**), there was a significant increase in the non-treated cells in group 2 (*p* < 0.05), when compared to the control in group 1, but the increase in the photobiomodulation-treated cells was not significant (*p* > 0.5). There was no clear difference between photobiomodulation-treated and non-treated cells. *(n* = 9 for each group). The comparison between the control group cell cultures (group 1; non-treated cells) and the photobiomodulation- and non-treated cells of group 2 is shown by the line above each of the columns; if the line finishes in-between the columns, then it reflects the same level of statistical significance. ±SEM, * *p* < 0.05, ** *p* < 0.01, *** *p* < 0.001.

**Figure 4:**
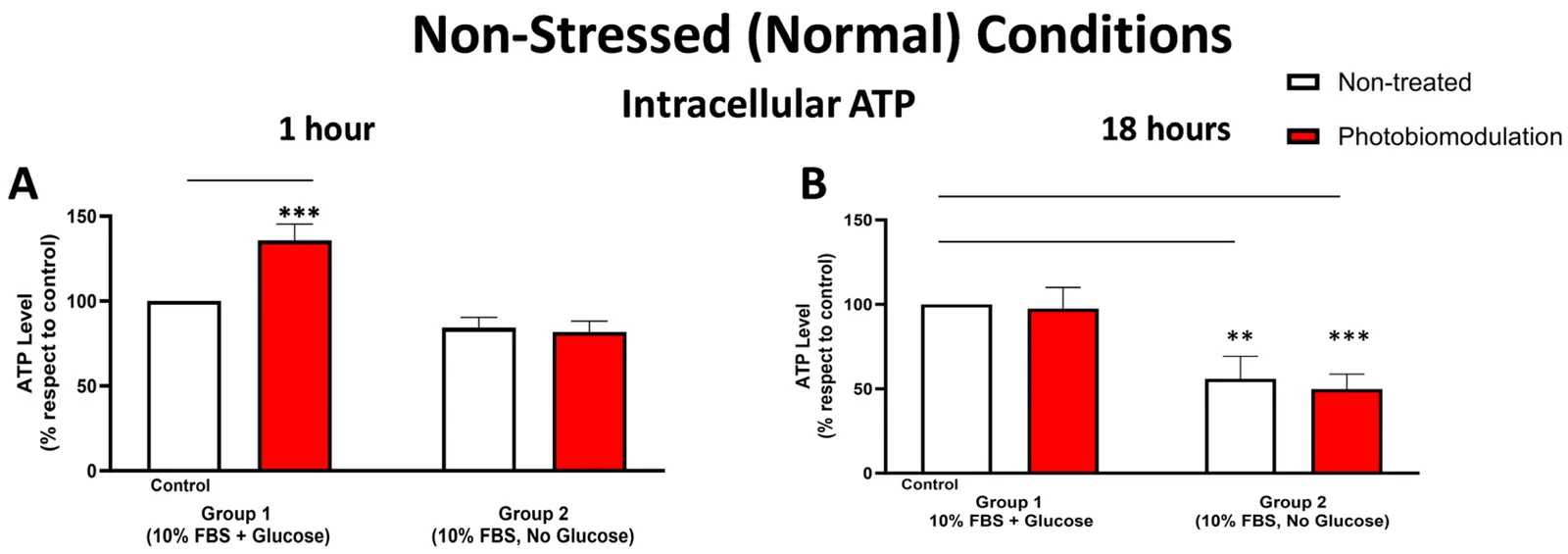
Graphs showing the level of ATP 1 h (**A**) and 18 h (**B**) after the last dose (or not) of photobiomodulation in non-stressed conditions. The levels of intracellular ATP in the cultures without glucose (group 2) were generally lower than the cultures with glucose (control cells in group 1) after 1 h post-treatment (**A**), even though that was not statistically significant. Compared to the control cells, the photobiomodulation-treated cells in group 1 (in red) had considerably higher levels of ATP (*p* < 0.001) (*n* = 12 for each group). After 18 h post-treatment (**B**), the levels of ATP in both the photobiomodulation-treated (in red) and non-treated cells (in white) in groups 2 were significantly lower than the control (*p* < 0.001 and *p* < 0.01, respectively). There was no significant difference between the photobiomodulation-treated cells and their respective controls (*p* > 0.5 for both groups; *n* = 9). ±SEM, ** *p* < 0.01 and *** *p* < 0.001.

**Figure 5:**
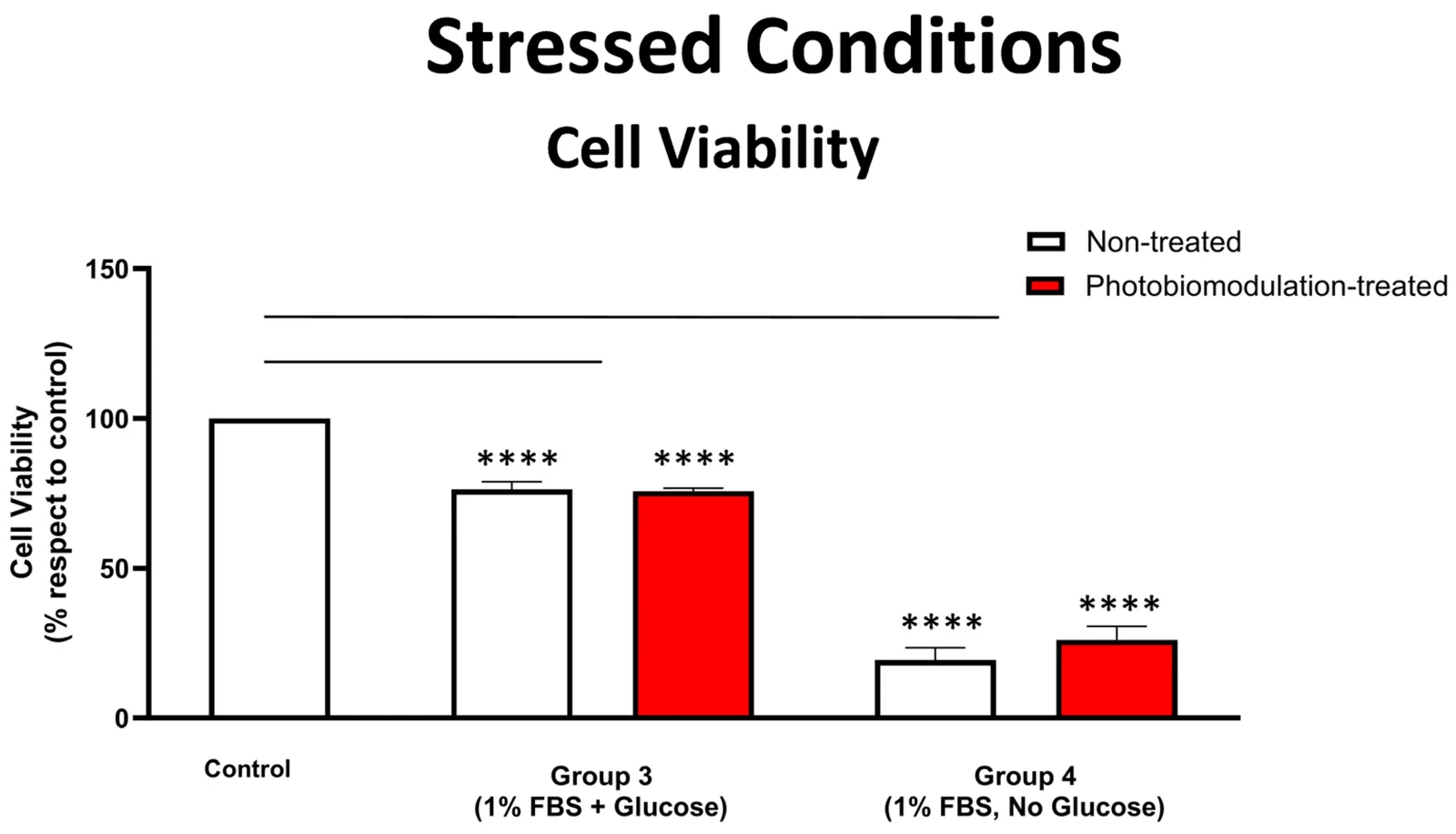
Graph showing the results of the cell viability test on the effect of photobiomodulation under stressed conditions. The MTS/PMS test was carried out with cells grown in media with 1% FBS, with either glucose (group 3) or without glucose (group 4). The results show that there were statistically significant decreases in both the photobiomodulation-treated (in red) and non-treated cells (in white) of both groups 3 (*p* < 0.0001) and 4 (*p* < 0.0001 for treated and non-treated cells), compared to the control. Further, photobiomodulation treatment had no effect on cell viability, indicating that the photobiomodulation treatment was not detrimental to the cells *(n* = 9 for each group). The comparison between the control group cell cultures (non-treated cells) and the photobiomodulation- and non-treated cells of group 3 and 4 is shown by the lines and the asterisks (*) above each of the columns; if the line finishes in-between the columns, then it reflects the same level of statistical significance. Non-significant comparisons are not indicated. Data are expressed as the mean percentage (%) of control values ± SEM, **** *p* < 0.0001.

**Figure 6:**
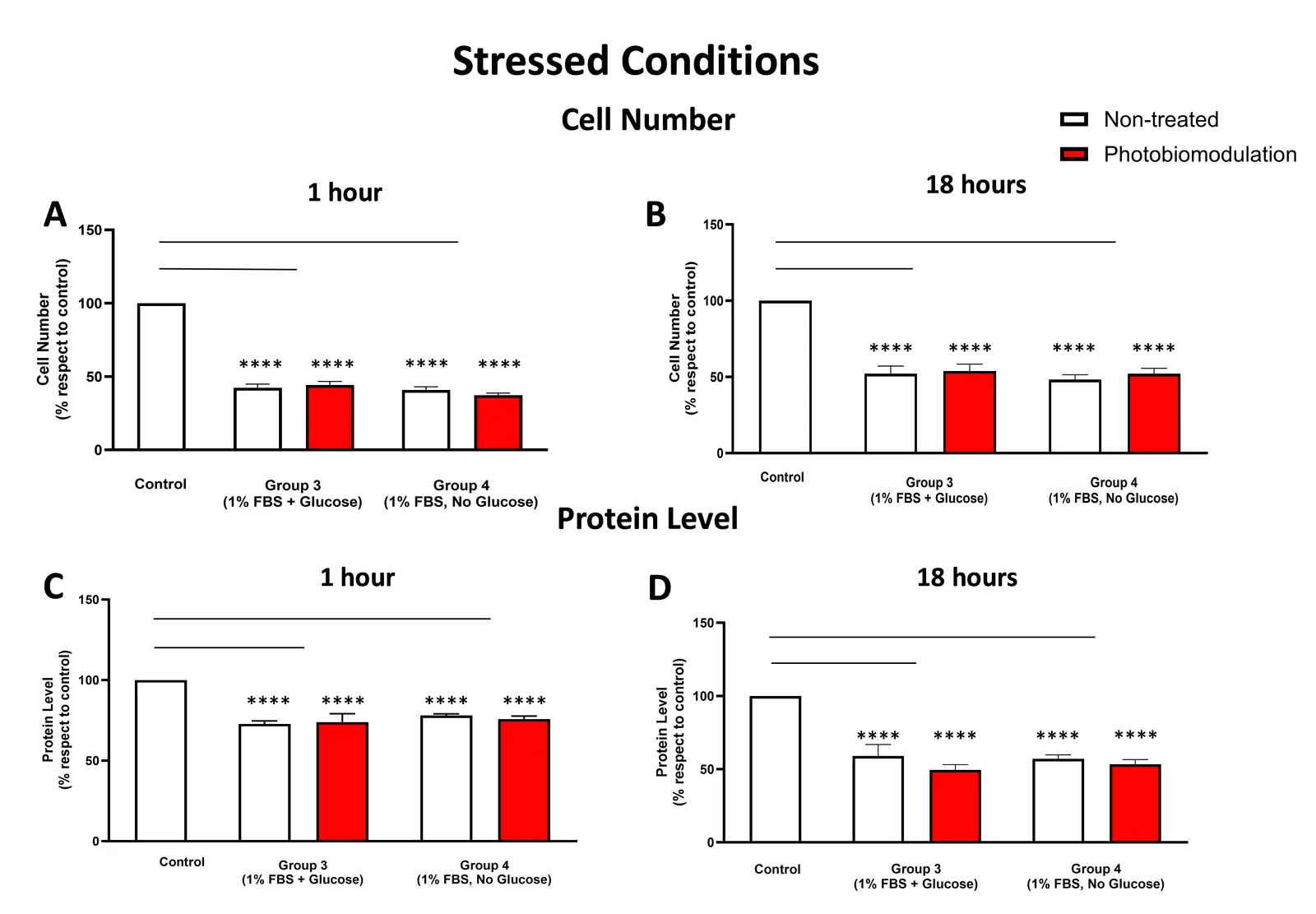
Graphs showing the impact of photobiomodulation on cell (number) proliferation in the stressed conditions, with either glucose or without glucose, within 1 h post-treatment (**A**). There were statistically significant reductions in the number of cells in both the non-treated (in white) and treated cells (in red) from groups 3 and 4, when compared to the control group (*p* < 0.0001 for both non-treated and treated cells of the two groups) (*n* = 9 in all groups). (**B**) shows the results of the cell (number) proliferation after 18 h post-treatment. Similar to the results obtained after 1 h treatment, groups 3 and 4 had significantly fewer cells (*p* < 0.0001 in both groups) in both the photobiomodulation–treated and non-treated cells compared to the control cells. No significant difference was evident between photobiomodulation-treated and non-treated cells in both groups (*p* > 0.1; *n* = 9). (**C**) Graph showing the level of protein within 1 h after the last application of photobiomodulation. Similar to cell number, there was a significant decrease in the protein level in cells from both photobiomodulation-treated and non-treated cells in groups 3 and 4 compared to the control (*p* < 0.0001 for both groups). No significant difference was evident between photobiomodulation-treated and non-treated cells for all groups (*p* > 0.5; *n* = 12). (**D**) Graph showing the level of protein within 18 h after the last application of photobiomodulation. There was a significant decrease in the protein level in cells in groups 3 and 4 compared to the control (*p* < 0.0001 for both groups). No significant difference was evident between photobiomodulation-treated and non-treated cells for all groups (*p* > 0.5; *n* = 9). The comparison between the control group cell cultures (non-treated cells) and the photobiomodulation- and non-treated cells of group 3 and 4 is shown by the lines and the asterisks (*) above each of the columns; if the line finishes in-between the columns, then it reflects the same level of statistical significance. Non-significant comparisons are not indicated. ±SEM, **** *p* < 0.0001.

**Figure 7:**
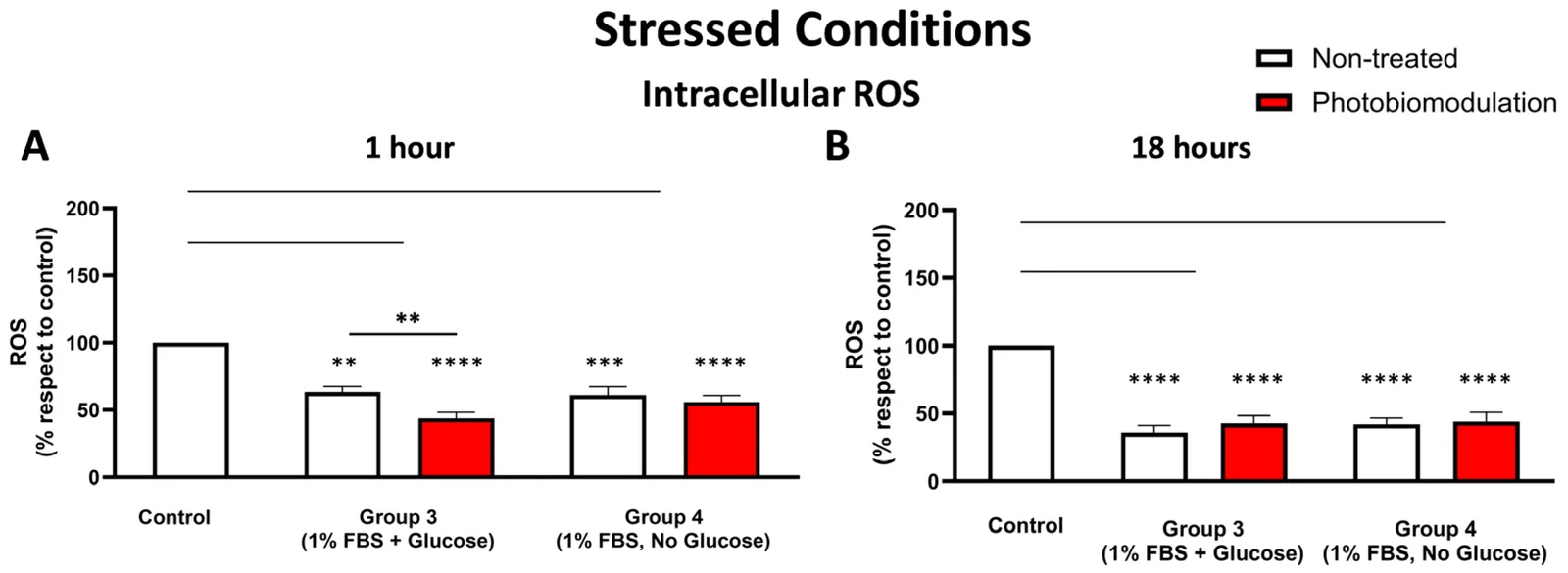
Cells grown under stressful conditions (1% FBS) with or without glucose were examined to evaluate the degree of cellular stress by measuring the level of ROS at 1 h (**A**) and 18 h (**B**) after the last dose of photobiomodulation. Both photobiomodulation-treated (in red) and untreated cells (in white) in groups 3 and 4 in graph (**A**) showed a significant decrease in the level of cellular ROS (*p* < 0.0001 for the photobiomodulation-treated, *p* < 0.0005 for non-treated cells for group 3, and *p* < 0.0001 for both photobiomodulation-treated and non-treated cells for group 4). In group 4, there was no significant difference between the non-treated and photobiomodulation-treated cells, whereas in group 3, there was a statistically significant difference between the non-treated and photobiomodulation-treated cells (*p* < 0.01; *n* = 12). The level of cellular ROS after 18 h (**B**) in groups 3 and 4 in both photobiomodulation-treated and non-treated cells was significantly decreased, when compared to the control (*p* < 0.0001 for both photobiomodulation-treated and non-treated cells in both group 3 and 4). There was no clear difference between photobiomodulation-treated and non-treated cells. (*n* = 9). The comparison between the control group cell cultures (group 1; non-treated cells) and the photobiomodulation- and non-treated cells of group 3 and 4 is shown by the lines and the asterisks (*) above each of the columns; if the line finishes in-between the columns, then it reflects the same level of statistical significance. Non-significant comparisons are not indicated. The comparison between the control group cell cultures (non-treated cells) and the photobiomodulation- and non-treated cells of group 3 and 4 is shown by the lines and the asterisks (*) above each of the columns; if the line finishes in-between the columns, then it reflects the same level of statistical significance. Non-significant comparisons are not indicated. ±SEM, ** *p* < 0.01, *** *p* < 0.001 and **** *p* < 0.0001.

**Figure 8:**
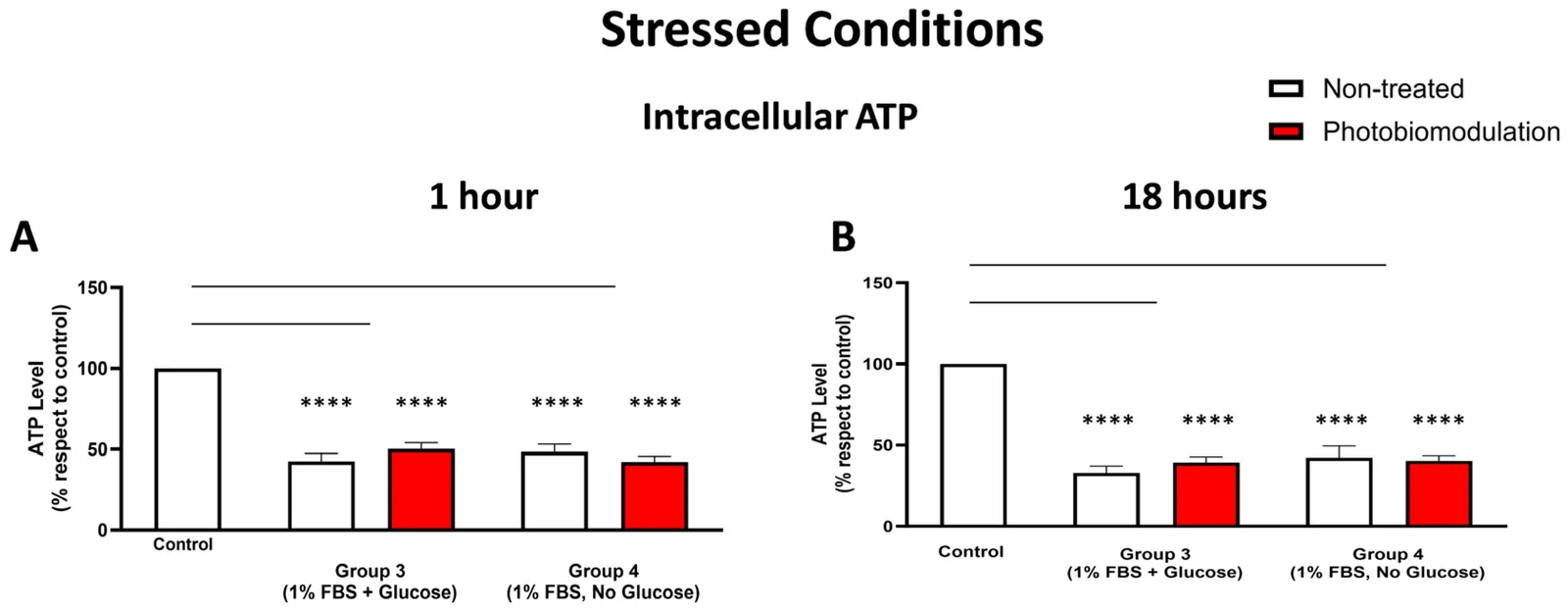
The level of intracellular ATP was measured in the stressed cells, and the results show that in comparison to the control group, the levels of ATP in both the photobiomodulation-treated (in red) and untreated cells (in white) decreased significantly within the 1 h after the last dose of photobiomodulation (**A**) and these reached significance for groups 3 (*p* < 0.0001; for both cell groups) and 4 (*p* < 0.0001; for both cell groups). There was no clear difference between non-treated and photobiomodulation-treated cells in both groups (*n* = 12). (**B**) shows the results of the 18 h post-treatment. The levels of cellular ATP in both the photobiomodulation-treated and non-treated cells in groups 3 and 4 were significantly lower than the control (*p* < 0.0001 for both cell groups and for both photobiomodulation-treated and non-treated cells). There was no significant difference between the photobiomodulation-treated cells and their respective controls (*p* > 0.5 for all four groups; *n* = 9). The comparison between the control group cell cultures (non-treated cells) and the photobiomodulation- and non-treated cells of group 3 and 4 is shown by the lines and the asterisks (*) above each of the columns; if the line finishes in-between the columns, then it reflects the same level of statistical significance. Non-significant comparisons are not indicated. ±SEM, **** *p* < 0.0001.

In the section that follows, our focus will be on the impact of photobiomodulation on fibroblast cells in the two main sets of experimental conditions: non-stressed (normal; Figure 1, Figure 2, Figure 3 and Figure 4) and stressed (Figure 5, Figure 6, Figure 7 and Figure 8), either with or without glucose. We will consider four measures, namely cell viability, cell proliferation and protein content, cell stress (intracellular ROS levels), and metabolic (intracellular ATP) activity at both 1 h (short-term) and 18 h (longer-term) after the last photobiomodulation treatment (or not).

### 3.1. Non-Stressed (Normal) Conditions

#### 3.1.1. Cell Viability

Figure 1 shows that cultures of fibroblast cells—both photobiomodulation- and non-treated—grown without glucose (group 2) had reduced viability and overall metabolic activity compared to the control group cell cultures grown with glucose (group 1; *p* < 0.0001). After the last treatment, there were no clear differences (*p* > 0.5) between the photobiomodulation-treated and non-treated cells of either group (group 1 and 2; Figure 1), indicating that at our doses, photobiomodulation—while not enhancing viability—was not toxic to the cells.

#### 3.1.2. Cell Number (Proliferation) and Protein Content

At both 1 h (Figure 2A) and 18 h (Figure 2B), the number of fibroblast cells—both photobiomodulation- and non-treated—grown without glucose (group 2) was significantly lower compared to the control group cell cultures grown with glucose (group 1; *p* < 0.0001). At both 1 h (Figure 2A) and 18 h (Figure 2B), there were no significant differences (*p* > 0.5) between the photobiomodulation- and the non-treated cells after the last treatment. The results of the cell proliferation tests were confirmed subsequently by measuring the protein levels. As for cell proliferation, cultures of cells at 1 h (Figure 2C) and 18 h (Figure 2D)—both photobiomodulation- and non-treated—that were grown without glucose (group 2) had a lower quantity of protein than the control group cell cultures grown with glucose, reaching statistical significance for the non-treated cells at 1 h (*p* < 0.01; Figure 2A) but not quite for photobiomodulation-treated cells at 1 h (*p* > 0.5; Figure 2A) and for both photobiomodulation- and non-treated cells at 18 h (*p* > 0.5; Figure 2B). Further, and importantly, there were no significant differences in the photobiomodulation-treated cells compared to the non-treated cells between the two groups of cell cultures at both 1 h (Figure 2A) and 18 h (Figure 2B) post-treatment (*p* > 0.5).

#### 3.1.3. Cellular Stress (ROS Levels)

At 1 h (Figure 3A) post-photobiomodulation treatment, there were clear increases in the level of intracellular ROS in the fibroblast cell cultures—both photobiomodulation-treated (*p* < 0.001) and non-treated (*p* < 0.01)—grown without glucose (group 2), when compared to the control group cell cultures (group 1). At 18 h post-photobiomodulation treatment (Figure 3B), the level of intracellular ROS in cell cultures grown without glucose (group 2) increased compared to the control group cell cultures (group 1), reaching significance for the non-treated cells (*p* < 0.05) but not quite for photobiomodulation-treated cells (*p* > 0.5). There were no clear differences (*p* > 0.5) between the photobiomodulation-treated and non-treated cells of both groups at both 1 h (Figure 3A) and 18 h (Figure 3B) after the last treatment.

#### 3.1.4. Metabolic (ATP) Activity

Figure 4A,B show that intracellular ATP levels were generally lower in the cell cultures—both photobiomodulation- and non-treated—grown without glucose (group 2) compared to the control group cell cultures with glucose (group 1); this decrease reached significance at 18 h (Figure 4B; *p* < 0.001 (photobiomodulation-treated) and *p* < 0.01 (non-treated)) but not at 1 h (Figure 4A; *p* > 0.1). When comparing the intracellular ATP levels between the photobiomodulation-treated and non-treated cells, there was a distinct (35%) and significant (*p* < 0.001) photobiomodulation-induced increase in intracellular ATP in the cell cultures grown with glucose (group 1) at 1 h post-treatment (Figure 4A); this difference was not evident in the cell cultures at 18 h (Figure 4B; *p* > 0.5).

At this point, we should draw attention to the finding that in the cell cultures without glucose, there was a general decrease in intracellular ATP—signalling a reduction in mitochondrial activity—but quite surprisingly, an increase in intracellular ROS (Figure 3). This increase may be reflective of the fact that mitochondria are not the only sources of ROS production; for example, peroxisomes and endoplasmic reticulum, which are involved in the oxidation of fatty acids and proteins (which are present in the 10% FBS), also produce ROS. Further, the increase in ROS in these cultures without glucose can be reflective of a decrease in the clearance of ROS; cells are unable to clear ROS because glucose is required for the activity of glycolysis and the pentose phosphate pathway, both of which are involved in ROS detoxification and lowering ROS levels [35,36].

### 3.2. Stressed Conditions

#### 3.2.1. Cell Viability

Figure 5 shows that in the stressed conditions of low levels of FBS (1%), fibroblast cells—both photobiomodulation- and non-treated—grown with glucose (group 3) and, in particular, those grown without glucose (group 4), had significantly reduced cell viability, when compared to the control group cell cultures (*p* < 0.0001), suggesting that our stressed condition of 1% FBS was effective. There were no clear differences evident between the photobiomodulation-treated and non-treated cells of both groups (Figure 5; *p* > 0.5).

#### 3.2.2. Cell Number (Proliferation) and Protein Content

At both 1 h (Figure 6A) and 18 h (Figure 6B), there were significant reductions in the cell number in the stressed cell cultures—both photobiomodulation- and non-treated—with either glucose (group 3; *p* < 0.0001) or without glucose (group 4; *p* < 0.0001), compared to the control group cell cultures. There were no significant differences between the photobiomodulation- and non-treated cells at both time periods (Figure 6A,B; *p* > 0.5). When compared to the control at both 1 h (Figure 6C) and 18 h (Figure 6D), there were also statistically significant decreases in the protein levels of stressed cell cultures with glucose (group 3; *p* < 0.0001) and without glucose (group 4; *p* < 0.0001), for both the photobiomodulation-treated and non-treated cells, compared to the control group cell cultures. Further, there were no significant differences in the protein level of the photobiomodulation-treated cells compared to the non-treated cells in each of the two groups (Figure 6C,D; *p* > 0.5).

#### 3.2.3. Cellular Stress (ROS Levels)

At 1 h post-treatment (Figure 7A), there were significant reductions in the intracellular ROS levels of stressed cell cultures—both photobiomodulation- and non-treated—either with glucose (group 3; *p* < 0.0001 (photobiomodulation-treated) and *p* < 0.01 (non-treated)) or without glucose (group 4; *p* < 0.0001 (photobiomodulation-treated) and *p* < 0.001 (non-treated)) compared to the control group cell cultures; similar patterns were evident at 18 h (Figure 7B; *p* < 0.0001). When comparing the intracellular ROS levels between the photobiomodulation- and non-treated cells, there was a clear (30%) and significant (*p* < 0.01) photobiomodulation-induced decrease in the stressed cell cultures grown with glucose (group 3) at 1 h post-treatment (Figure 7A) but not at 18 h (Figure 7B; *p* > 0.1).

#### 3.2.4. Metabolic (ATP) Activity

There was a reduction in the intracellular ATP levels in the stressed cultures of fibroblast cells—both photobiomodulation- and non-treated—grown with glucose (group 3; *p* < 0.0001) and without glucose (group 4; *p* < 0.0001), when compared to the control group cell cultures both at 1 h (Figure 8A) and 18 h (Figure 8B) after the last treatment. There were no major differences in the intracellular ATP levels between the photobiomodulation-treated and non-treated cells in any of the stressed cell cultures (groups 3 and 4; *p* > 0.1 for 1 h and *p* > 0.5 for 18 h).

In summary, our results show that the stressed conditions of low levels of FBS, either with (group 3) or without glucose (group 4), had the clearest and most consistent decreases in measures of cell viability, cell proliferation and protein, intracellular ROS, and intracellular ATP levels in the cultured fibroblast cells. There was no clear and consistent photobiomodulation effect in the majority of our measurements, namely, cell viability, cell proliferation, and protein levels. There was, however, a clear photobiomodulation-induced effect evident in the intracellular levels of ATP and ROS. This effect appeared only in the cell cultures that included glucose, that is, group 1 for intracellular ATP (Figure 8A) and group 3 for intracellular ROS (Figure 8B).

## 4. Discussion

We have three key findings: photobiomodulation at the dose regime we used was (1) not cytotoxic, (2) not effective in enhancing cell viability and proliferation, together with protein levels, and (3) effective in changing ATP and ROS levels, and this was—most importantly—only in cultures where glucose was readily available. In the discussion that follows, we focus on these key findings and the effect of photobiomodulation on our fibroblast cell cultures under the different conditions.

### 4.1. Photobiomodulation Was Not Detrimental to Cell Survival

One feature that was clear from all of our measures, but particularly from the cell viability test, was that the dosage of the photobiomodulation treatment at 1.5 mW/cm^2^ for 120 s twice a day over 24 h for 2 days was not detrimental to the fibroblast cells; this dosage regime equated to 0.72 J/cm^2^. Many previous in vitro studies have also reported that photobiomodulation is not detrimental to cells, whether they be fibroblasts [37,38,39,40,41,42] or neurones [43,44,45], across a wide range of doses, for example from 0.3 to 50 J/cm^2^. Our dosage regime of 0.72 J/cm^2^ was within this range and confirms the widely held view that photobiomodulation is an entirely safe treatment option, with little or no evidence for cytotoxicity, across an extensive range of therapeutic dose applications [2].

### 4.2. Photobiomodulation Did Not Enhance Most Measures of Cell Function and Survival

In this study, we chose to use the photobiomodulation dosage regime of 0.72 J/cm^2^ in the fibroblast cell cultures, one that has been used previously using red (660 nm) and near infrared wavelengths (980 nm) [31]. We found that at both 1 h and 18 h after the last photobiomodulation treatment, there was no effect on many of our measures—namely, cell viability, cell proliferation, and protein levels—in both our non-stressed and stressed cell culture conditions. Previous studies have, using a range of treatment schedules and wavelengths, reported similar patterns on fibroblast cells. For example, following applications of either ~8 J/cm^2^ [40,41,42] or 24 J/cm^2^ [46], photobiomodulation was reported to have no impact on the cell viability of fibroblast cells up to 24 h after the last treatment (using 635 nm and 600 nm). Indeed, only after the much higher dose of 50 J/cm^2^ (using 670 nm), and after an extended survival period of 50 h after the last treatment, has there been any evidence of a photobiomodulation-induced increase in fibroblast cell proliferation [38]. Hence, it appears that only at the very higher dosage regimes and longer survival periods does photobiomodulation help boost cell survival in cultures. We should also note that the wide range of wavelengths across the red to near infrared range used by these previous studies may well have also contributed to different cellular outcomes after photobiomodulation treatment; these different wavelengths, although largely targeting mitochondrial activity, may also have other intrinsic targets within the cells, such as heat-gated calcium ion channels and transient potential receptor ion channels (e.g., [16,47]). For our purposes here, using the lower dose regime [31] at 810 nm near infrared light wavelength, we did detect a clear photobiomodulation-induced effect, at least in the key measures of ATP and ROS levels (see below). Indeed, previous studies have treated cell cultures with 808 nm or 810 nm wavelengths and recorded changes in both ATP and ROS levels [45,47,48,49].

### 4.3. Photobiomodulation Was Effective Only with ATP and ROS Levels and When Glucose Was Available

Our most striking finding was that photobiomodulation was effective only in changing the measures of ATP and ROS levels; further, and most notably, these photobiomodulation-induced changes occurred only when there was glucose readily available. We found a clear difference between photobiomodulation- and non-treated cells in the non-stressed normal condition for ATP levels (group 1; ~35%) and in one of the stressed conditions for ROS levels (group 3; ~30%). For both of these measures, the cell cultures contained glucose; the corresponding cultures that did not contain glucose in the series (groups 2 and 4) did not show an increase in ATP levels nor a decrease in ROS levels after photobiomodulation treatment (Figure 3, Figure 4, Figure 7 and Figure 8). It is also noteworthy that this increase in ATP levels and decrease in ROS levels in the groups that contained glucose was evident only 1 h after the last dose of photobiomodulation and not 18 h. Hence, the effect was a short-term one.

Previous studies have reported similar photobiomodulation-induced effects in cell culture, in particular, an increase in ATP [46,50,51] and a decrease in ROS levels (e.g., [52,53,54,55]). Further, many studies have reported that the resultant changes in ATP and ROS levels are rather short-term but nevertheless, beneficial to cell function and survival (see Introduction and Conclusions [2,15,16]. Most relevant to our results here is that these effects were reported by previous studies using animals and cell cultures that contained glucose. As we have shown in the present study, these photobiomodulation-induced effects vanished when glucose was not available. It should be noted that other sources of metabolic substrates, such as lactate and pyruvate, may also generate similar photobiomodulation-induced improvements in cells [56].

## 5. Conclusions

In summary, our results indicate that photobiomodulation was effective in prompting a short-term increase in ATP energy and decrease in ROS levels and that these key changes occurred only when there was glucose available for the mitochondria to work with. The cellular benefits of the photobiomodulation-induced increase in ATP and decrease in ROS levels can be considerable; the increase in ATP can result in more readily available energy to drive intrinsic cell functions, while the decrease in ROS levels can offset any potential further cell dysfunction and damage [2,15,16]. Our findings lay a template for further explorations into the effect of photobiomodulation treatment—with and without the availability of glucose—in cell cultures of neurones and astrocytes, as well as in vivo experiments on animal models of disease. Taking it a step further, these first-up findings have considerable experimental and clinical implications; for optimal outcomes, photobiomodulation could be applied when glucose is readily available, in particular, soon after meals [57].

## Data Availability

All data are available from the corresponding author upon a reasonable request.
