# Correction: Hoh Kam, J.; Mitrofanis, J. Glucose Improves the Efficacy of Photobiomodulation in Changing ATP and ROS Levels in Mouse Fibroblast Cell Cultures. *Cells* 2023, *12*, 2533

**DOI:** 10.3390/cells15201854

**Published:** 2026-10-13

**Authors:** Jaimie Hoh Kam, John Mitrofanis

**Affiliations:** 1Fonds de Dotation Clinatec, https://ror.org/02rx3b187Grenoble Alpes University, 38000 Grenoble, France; 2Institute of Ophthalmology, https://ror.org/02jx3x895University College London, London EC1V 9EL, UK

## Missing Citation

In the original publication [1], ref. “Sharma, S.K.; Kharkwal, G.B.; Sajo, M.; Huang, Y.Y.; De Taboada, L.; McCarthy, T.; Hamblin, M.R. Dose response effects of 810 nm laser light on mouse primary cortical neurons. *Lasers Surg. Med.* **2011**, *43*, 851–859.” was not cited. The citation has now been inserted in the first paragraph of Section 2.2 and should read as follows:

Cell cultures were exposed to a diode laser emitting continuous near infrared light (λ = 810 nm) with a beam diameter of 2 mm (Ti-sapphire Laser). The laser was positioned on top of the polystyrene transparent cell culture six-well plate (13 *×* 10 cm), at a distance of approximately 10 cm. Following Sharma and colleagues [34], who used an 810 nm laser to treat neurones at fluences from 0.03–30 J/cm^2^ on cortical neurones and obtained cellular responses from 0.3 J/cm^2^, the dose here was 1.5 mW/cm^2^ (measured at the level of the cells in the dish) for 120 s (total of 0.72 J/cm^2^), and the cells were light-treated twice per day (morning and afternoon) over 24 h for two days. To prevent nuisance variables from other light sources, laser exposure took place in the dark.

## Text Correction

There was an error in the original publication. The value 15 mW/cm^2^ should be changed to 1.5 mW/cm^2^ and 7.2 J/cm^2^ should be changed to 0.72 J/cm^2^.

A correction has been made to the Abstract:

In this study, we tested the idea that photobiomodulation—the application of red to near infrared light (~λ = 600–1300 nm) to body tissues—is more effective in influencing cell metabolism when glucose is readily available. To this end, we used a mouse fibroblast (L-929) cell culture model and had two sets of conditions: non-stressed (10% FBS (foetal bovine serum)) and stressed (1% FBS), both either with or without glucose. We treated (or not) cells with photobiomodulation using an 810 nm laser at 1.5 mW/cm^2^ (~0.72 J/cm^2^).

A correction has been made to the first paragraph of Section 2.2:

Cell cultures were exposed to a diode laser emitting continuous near infrared light (λ = 810 nm) with a beam diameter of 2 mm (Ti-sapphire Laser). The laser was positioned on top of the polystyrene transparent cell culture six-well plate (13 *×* 10 cm), at a distance of approximately 10 cm. Following Sharma and colleagues [34], who used an 810 nm laser to treat neurones at fluences from 0.03–30 J/cm^2^ on cortical neurones and obtained cellular responses from 0.3 J/cm^2^, the dose here was 1.5 mW/cm^2^ (measured at the level of the cells in the dish) for 120 s (total of 0.72 J/cm^2^), and the cells were light-treated twice per day (morning and afternoon) over 24 h for two days. To prevent nuisance variables from other light sources, laser exposure took place in the dark.

A correction has been made to the paragraph in Section 4.1, the value 3 has been changed to 0.3 and corrected paragraph appears below:

One feature that was clear from all of our measures, but particularly from the cell viability test, was that the dosage of the photobiomodulation treatment at 1.5 mW/cm^2^ for 120 s twice a day over 24 h for 2 days was not detrimental to the fibroblast cells; this dosage regime equated to 0.72 J/cm^2^. Many previous in vitro studies have also reported that photobiomodulation is not detrimental to cells, whether they be fibroblasts [37–42] or neurones [43–45], across a wide range of doses, for example from 0.3 to 50 J/cm^2^. Our dosage regime of 0.72 J/cm^2^ was within this range and confirms the widely held view that photobiomodulation is an entirely safe treatment option, with little or no evidence for cytotoxicity, across an extensive range of therapeutic dose applications [2].

A correction has been made to the first sentence of the paragraph in Section 4.2:

In this study, we chose to use the photobiomodulation dosage regime of 0.72 J/cm^2^ in the fibroblast cell cultures, one that has been used previously using red (660 nm) and near infrared wavelengths (980 nm) [31].

## References

34. Sharma, S.K.; Kharkwal, G.B.; Sajo, M.; Huang, Y.Y.; De Taboada, L.; McCarthy, T.; Hamblin, M.R. Dose response effects of 810 nm laser light on mouse primary cortical neurons. *Lasers Surg. Med.* **2011**, *43*, 851–859. http://doi.org/10.1002/lsm.21100.

37. Fuchs, C.; Schenk, M.S.; Pham, L.; Cui, L.; Anderson, R.R.; Tam, J. Photobiomodulation Response from 660 nm is Different and More Durable than That from 980 nm. *Lasers Surg. Med.* **2021**, *53*, 1279–1293. https://doi.org/10.1002/lsm.23419.

Fuchs et al., 2021 is moved from citation [34] to [37]. With this correction, the order of some references has been adjusted accordingly. The authors state that the scientific conclusions are unaffected. This correction was approved by the Academic Editor. The original publication has also been updated.
